# The Use of Satellite Tags to Redefine Movement Patterns of Spiny Dogfish (*Squalus acanthias*) along the U.S. East Coast: Implications for Fisheries Management

**DOI:** 10.1371/journal.pone.0103384

**Published:** 2014-07-28

**Authors:** Amy E. Carlson, Eric R. Hoffmayer, Cindy A. Tribuzio, James A. Sulikowski

**Affiliations:** 1 Marine Science Center, University of New England, Biddeford, Maine, United States of America; 2 National Marine Fisheries Service Mississippi Laboratories, Southeast Fisheries Science Center, Pascagoula, Mississippi, United States of America; 3 National Marine Fisheries Service Auke Bay Laboratories, Alaska Fisheries Science Center, Juneau, Alaska, United States of America; University of California Davis, United States of America

## Abstract

Spiny dogfish *(Squalus acanthias)* are assumed to be a highly migratory species, making habitual north-south migrations throughout their northwestern Atlantic United States (U.S.) range. Also assumed to be a benthic species, spiny dogfish stock structure is estimated through Northeast Fisheries Science Center (NEFSC) bottom-trawl surveys. Recent anomalies in population trends, including a recent four-fold increase in estimated spawning stock biomass, suggest alternative movement patterns could exist for this shark species. To obtain a better understanding of the horizontal and vertical movement dynamics of this species, Microwave Telemetry pop-up satellite archival X-Tags were attached to forty adult spiny dogfish at the northern (Gulf of Maine) and southern (North Carolina) extents of their core U.S. geographic range. Reconstructed geolocation tracks ranging in lengths from two to 12 months suggest that the seasonal migration patterns appear to be local in nature to each respective northern and southern deployment site, differing from previously published migration paradigms. Differences in distance and direction traveled between seasonal geolocations possibly indicate separate migratory patterns between groups. Kernel utilization distribution models also suggest strong separate core home ranges. Significant differences in seasonal temperature and depths between the two regions further substantiate the possibility of separate regional movement patterns between the two groups. Vertical utilization also suggests distinct diel patterns and that this species may not utilize the benthos as previously thought, potentially decreasing availability to benthic gear.

## Introduction

Understanding both large (i.e. migratory) and small (i.e. diel) scale movement patterns are essential components of successful fisheries management plans, especially for species that are considered highly migratory and/or are commercially harvested [1]–[6]. Until recently, most of what is known about the movement of fishes has been collected from conventional (e.g. plastic streamer/dart) tagging studies, reliant on fisheries dependent recoveries [7]–[9]. When tags are recovered, they reveal only the net distance traveled and time elapsed between tagging and recovery location (see Kohler & Turner [10] for a review of shark studies). While this information has provided scientists with a wealth of knowledge concerning straight line movement patterns, the actual movement path of the fish, including the depths visited, remained unknown [5]–[6], [11]. The abundance and distribution of many marine fish species in United States (U.S.) coastal waters is measured by fisheries-independent surveys conducted by National Marine Fisheries Service (NMFS), various universities, and state agencies. While these surveys have supplied critical datasets for ongoing stock assessments and management plans, they sample a large spatial scale, usually with a single gear type, and a restricted temporal scale (i.e. once or twice per year), only providing a limited spatial and temporal snapshot of the stock structure [12]–[14].

Pop-up satellite archival tags (PSATs), a relatively new technology, can provide information on horizontal and vertical movements of fishes in relation to conditions and/or features of their biophysical environment. Unlike traditional satellite tags, which actively track an animal's location only when the tag breaks the ocean surface and communicates with orbiting satellites, PSATs have the ability to estimate position from light intensity while remaining completely submerged [15], making them suitable for animals that do not surface often or at all. The PSAT is preprogrammed to detach from the animal on a specific date and to transmit stored data to a passing satellite. Thus the “recovery” of the tag, or rather the tag data, is fisheries independent, as the tag does not need to be physically recaptured for the data to be recovered [11], [16]. Despite the downsides of PSATs, including positional inaccuracies, premature pop-off, and high expense per unit [17], these tags have allowed scientists to successfully collect data that were previously unavailable to them, such as determining salmon shark (*Lamna ditropis*) niches [18], depth preferences and spawning grounds of Atlantic bluefin tuna (*Thunnus thynnus*) [19]–[20] and migration patterns of white sharks (*Carcharodon carcharias*) [21]–[24].

In the northwest Atlantic Ocean, the spiny dogfish (*Squalus acanthias*) is a ubiquitous species ranging from Newfoundland to Florida. This species is most abundant from the Gulf of Maine (GOM) to North Carolina (NC) where they are managed as one unit stock [25]–[29]. Early studies based on NMFS Northeast Fisheries Science Center (NEFSC) bottom-trawl surveys [29] and conventional tag and recapture studies [8] suggested that spiny dogfish were highly migratory, making synchronous, coast-wide seasonal movements southward from the GOM to NC in the autumn, and back to the GOM from NC in the spring [26]–[30].

Captured in large quantities in multi-species NEFSC bottom-trawl surveys, spiny dogfish are considered a benthic species in which subsequent annual biomass estimates are derived [28], [30]. Based on these surveys, spiny dogfish were considered to be the most abundant shark species in U.S. waters until the late 1980's, when a substantial decrease in spawning stock biomass (SSB) occurred, reducing this biomass value from approximately 234,000 mt to approximately 52,000 mt (1991–1999) [31]–[34]. A rebuilding plan consisting of a 1,814 mt annual quota [35] and reduced possession limits for vessels fishing in federal waters was established in 2000 with the aim to increase the SSB above threshold levels (45,000 mt). Despite these measures, the “K” selected life history characteristics (i.e. slow growth and late maturity) of spiny dogfish were thought to preclude the stock from rebuilding before 2020 [32], [36]. However, biologically implausible increases in SSB [37]–[38] resulted in a rebuilt stock status as early as in 2008 [39]. Consequently, the stock was declared rebuilt and SSB estimates continued to increase, catch limits were also steadily raised from approximately 1,814 mt (2008) to 18,506 mt (2013) [40]–[42].

Recent information suggests that the anomalies and uncertainties in the SSB could be partially attributed to spiny dogfish horizontal and vertical movement patterns. For example, a preliminary study by Sulikowski et al. [43], which tested the efficacy of Microwave Telemetry X-Tags (Microwave Telemetry, Inc., Columbia, MD, USA), tracked the movement of three PSAT tagged female spiny dogfish. The preliminary results suggested that these sharks did not make long distance migrations over the deployment period, but rather moved in an easterly direction towards offshore waters. Diel vertical movement patterns suggested that the sharks were highly active during both the day and night, spending a portion of time off-bottom and likely out of reach of the trawl survey nets. The information from Sulikowski et al. [43] coupled with studies by Rulifson & Moore [14] and Rulifson et al. [8] corroborates that that spiny dogfish widely segregate by sex and size [31], particularly in the core GOM to NC areas. These observations could, in part, start to explain some of the anomalies in the SSB estimates.

Given the uncertainties in stock estimates for this species and the potential vertical behavioral patterns that could possibly, limit the effective capture of spiny dogfish by NEFSC bottom-trawl surveys, the goals of the current study were to: (1) deploy satellite tags on spiny dogfish at either extent of their core range (GOM and NC), (2) determine whether migration patterns are in unison, synchronous and/or separate; (3) determine whether regional, seasonal, and diel differences exist in the vertical activity of the sharks between the two sample populations; and (4) compare the vertical and horizontal movement patterns of satellite tagged spiny dogfish to the NEFSC bottom-trawl survey over the same spatiotemporal period.

## Methods

Tag and release activities were conducted in federal waters and no specific agency permissions were required. This protocol was approved by the University of New England Institutional Animal Care and Use Committee (IACUC) under permit number UNE011-2009. A total of 40 pop-up satellite archival X-Tags (PSATs) were deployed on adult (males> = approximately 68 cm total length (TL), females> = approximately 88 cm TL [44]) spiny dogfish from 2009 to 2011. The X-Tags weighed 40 g in air, measured 120 mm in body length, 185 mm in antenna length, and 32 mm in maximum diameter. Twenty tags were deployed on spiny dogfish in the GOM (43°32.542N, 70°00.897W) during late summer and autumn to capture a representative sample of individuals before they headed south for their presumed seasonal migration. These individuals were all caught off the University of New England's 7.7 m R/V *Llyr*, using hook-and-line fishing methods on 7/0 Sea Wolf cod rigs baited with herring (*Clupea harengus*). An additional twenty spiny dogfish were tagged in spring off the coast of NC (35°45.549N, 75°29.035W), just prior to their presumed northward migration. These individuals were captured using a 300 m×3 m gillnet with 9 cm mesh off of the F/V *Tarbaby*. An effort was made to deploy tags on an equal number of male and female spiny dogfish in each region. While the dogfish used were not weighed individually, adult dogfish range in weight from 7.3–10 kg [45]–[46] and subsequently, those tagged were presumed large enough to carry the tag, based on the 2% tag to body weight ratio rule of thumb [47] and unpublished observations on dummy-tagged captive spiny dogfish [J. Sulikowski, pers. comm.]. All future references to “northern” spiny dogfish were those tags deployed in the GOM and “southern” spiny dogfish were those tags deployed in NC.

The X-Tags were affixed to each spiny dogfish by attaching a tether to the second dorsal fin spine. A 1.5 mm hole was drilled through the second dorsal spine, and attached with a lightweight tether comprised of two stainless steel nicopress crimp sleeves and 20 cm length of 1.6 mm diameter, 136 kg test high quality monofilament Jinkai fishing line. The entire attachment was encased in a 7.5 cm length of 3.2 mm inner diameter×6.4 mm outer diameter Nalgene silicone tubing, ensuring that the crimps used to secure the monofilament did not come within 2.5 cm of the detachment mechanism on the tag (as specified by the manufacturer). Spiny dogfish were then placed within a 2 m×2 m×4 m on deck live well to recover. After 30 min, spiny dogfish deemed suitable for use in the study (i.e. actively moving in the tank), were released back into the ocean.

All X-Tags were programmed to pop-up 12 months after deployment, and were set to collect daily light levels (up to <4×10^−5^ Lux @ 555 nm), depth (range 0 to 1296 m; resolution ±5.4 m), and temperature (range −4 to +40°C; resolution ±0.23°C). Temperature and depth data were recorded at 2-min intervals and were available at that resolution for tags which were physically recovered. For tags that were not physically recovered, lower resolution compressed data were recovered via satellite transmissions. The data compression (i.e. resolution) was dependent on the length of deployment. Resolution of data can be available at 15-min increments (for tag deployments up to four months), 30-min increments (four–eight month deployments) or 60-min increments (eight–twelve month deployments). The X-Tag data compression programming for transmitted data also has limits on the rate of temperature and depth changes it can record. Limits of X-Tag recorded depths are constrained to a change in descent between recordings of 166.8 m and ascent of 172.1 m, known as delta-limited values, thus the actual depth of a delta limited dive/ascent is unknown. Changes in depth that exceeded these limits accounted for on average 0.76±0.1% (descent) and 0.61±0.1% (ascent) of the values recorded per individual and were removed from analyses. Temperature records were not affected by delta-limited values. These limitations do not extend to data stored on the tag for those physically recovered. The X-Tags were also programmed with a constant depth sensor in the event the tag records a constant depth (within 3 m) for six days that tag would detach and start reporting to the satellite. Thus, if the animal dies and sinks to the bottom or the tag detaches and floats at the surface for more than six days, the tag begins to transmit the archived data. Additionally, prior to attachment, X-Tags were tested for pop-up mechanism and satellite transmission, as according the manufacturer's instructions. Summarized data were transmitted through the ARGOS satellite array back to Microwave Telemetry, where the raw data (i.e. light levels, pressure, temperature archives) were compiled and preliminary daily geolocations were approximated using a proprietary algorithm.

Once these data were received, they were processed through a stepwise set of filters and analyses. The preliminary estimated geolocations were fitted with a state space extended Kalman filter model (kftrack in analyzepsat 3.0 package) [48]–[49] within the R v2.10.1 statistical language environment [50] to produce the most statistically probable movement track, following modified methods from Sulikowski et al. [43]. In the event that the tag detached early, pop-up date was determined by signs of detachment in the temperature and depth data (i.e. sustained time at the surface combined with unusually warm [summer] or cold [winter] temperatures) and the track was terminated on that date. The most probable track was then bathymetrically corrected (btrack in analyzepsat 3.0 in R) to increase accuracy by comparing randomly sampled known bathymetry locations within the CIs to the maximum daily depths recorded on the tags [51]–[54]. The bathymetrically filtered geolocations were also accompanied by a 95% confidence interval (CI) around each point to account for error in the geolocation calculations. The bathymetric correction is particularly useful for estimating tracks based on limited light levels, few latitude estimates, and increased time at depths deeper than 200 m, which occurred for the majority of tagged spiny dogfish. An additional sea-surface temperature filter (ukfsst in analyzepsat 3.0 package in R) was also applied to the data; however, with the majority of time at liberty spent at depth, was unsuccessful and therefore not used in the analysis [17], [55]–[58]. The geolocations resulting from the bathymetric correction were considered the most probable geolocations and were the positions used in all subsequent analysis. Although no direct sex differences were analyzed, geolocations for mature females (> = 88 cm TL [44]) for the months October–May (months when parturition is known to occur [28], [59]–[60], [62]–[65]) were mapped separately from the rest of the tags.

The bathymetrically corrected geolocation points were used to examine magnitude and direction of movements as well as habitat use. To do so, the direction and magnitude between individual points were measured for each individual in both regions. The calculations were then summarized as circular histograms for each season and separated by region. Seasons were classified as winter (December–February), spring (March–May), summer (June–August), and autumn (September–November). The bathymetrically corrected points were also used to calculate kernel utilization distributions. Utilization distributions (UDs) were calculated (using R adehabitat package, least squares cross validation method) from these approximated tracks to yield utilization (similar to home range) density gradients, as a continuous likelihood coverage from 0–100% calculated around the geolocation points. These UDs were produced for each individual and binned groups, including the whole tag group (inclusive of all sharks from both tagging sites), separate northern released tags only (19 successful northern tags), and southern released tags only (15 successful southern tags). To look for possible migratory patterns, UDs were also calculated by season for both the northern and southern groups. Once all these UD gradients were produced, 95% and 50% usage contours were extracted out from the gradients to find the total usage space UD (95% usage) and core usage space UD (50% usage). The 95% confine represents total usage space, or the animals' entire activity space used for normal activities [66] during the duration of tracking, including erroneous points and anomalous behavior [67]. The 50% confine represents core usage space and is highlighted by selected areas of concentrated use that has greater significance to the animal than other sites within the home range [67]. Geolocation points (results from bathymetric correction) with CIs were compared to recreated maps of temporally appropriate NEFSC spring and autumn bottom-trawl survey sampling stations. These surveys begin off NC and work northward to the GOM following a stratified random design within different geographic strata [68]–[70]. Station bottom temperature collected during NEFSC trawl surveys [71]–[75] from autumn 2009 to spring 2012 were interpolated in ArcGIS 9.3 to create spatially continuous datasets between survey stations. These interpolations were then visually compared to tag geolocations to determine if any association of movement patterns or distribution existed seasonally (for seasons in which the surveys occurred, spring and autumn) and monthly (for months in which the surveys occurred, which varied in both seasons from 2009 to 2012) [71]–[75]. The terms “seasonally” and “monthly” are not meant to necessarily imply all seasons or months were used in the comparisons. Exact start and stop dates of the biannual surveys were used with exact geolocation timestamps to avoid under- or over-estimation. Areas of overlap between survey stations and geolocations (including CIs) were identified to determine the percentage of geolocations located within the temporally corresponding survey locations, as previously described, using only the stations and geolocations with the same timestamp. Of the geolocations that were possibly “available” (to be captured) by the bottom-trawl surveys, Overlap in satellite tag depths and the temporally corresponding bottom-trawl set depths were compared. To do so, possible spiny dogfish depth ranges were calculated from each individual time stamped tag recorded depth, including vertical tag error of ±2.5 m. Temporally simultaneous trawl depth ranges (trawl set depth plus height of the trawl's headrope, +4 m, to account for vertical spread [70]) were also calculated to find the vertical span of bottom covered by the trawl net. Instances where the tag and trawl depth ranges overlapped were considered to be likely available to be captured. For the remainder of this paper, the term “availability” refers to the availability of the tagged spiny dogfish (based on the ensuing results) to be captured in the bottom-trawl survey, in the total combined horizontal and vertical planes.

Depth and temperature data were evaluated on an individual and binned groupings basis, following the same scheme as the horizontal data. All means were reported with plus/minus standard error (± SE). Overall differences between northern and southern temperature and depth data were tested for statistical significance using a t-test in SigmaPlot (Systat Software, San Jose, CA). Seasonal (temperature and depth) and diel (depth) differences between the two groups were tested for statistical significance using a two-way Analysis of Variance (ANOVA). Days that did not have a full daily record (96 recordings at 15 min intervals, 48 recordings at 30 min intervals, or 24 recordings of 60 min intervals) of temperature and depth were excluded when analyzing diel movement patterns. Timestamps were also converted from Coordinated Universal Time (UTC) to Eastern Standard Time (EST), where values between 08:00–20:00 were binned as day, and between 20:00–08:00 binned as night. Diel depth was evaluated for the northern and southern groups overall, as well as seasonal differences between the groups. Diel depth patterns were assessed by calculating the difference between the depth at time of day and the daily mean depth for each individual spiny dogfish, then averaged by time of day for the two groups.

## Results

A total of 20 X-Tags were deployed in the northern group (TL range = 68–100 cm; males n = 10, mean TL: 76.3±2.4 cm, females n = 10, mean TL: 88.4±1.7 cm) and were released on four separate events, October 2, 2009 (n = 9), November 9, 2009 (n = 1), July 17, 2010 (n = 6), and August 13, 2010 (n = 4). Tags deployed in the southern group (TL range = 74–104 cm) were attached to nineteen females (mean TL 92.6±1.4 cm) and one male (TL 74 cm) on April 13, 2011. Of the 40 deployed X-Tags on spiny dogfish, 34 (northern = 19, southern = 15) detached from the sharks and transmitted data successfully (Table 1). In addition, three tags from each deployment site were also physically recovered and returned for high-resolution data extraction. Tags from both regions (northern = 1, southern = 5) did not transmit data. Days at liberty for both regions were comparable with a mean duration of 196 days (±29) in the northern group and 207 days (±30) in the southern group. The geolocation points used in the subsequent analysis all represent daily locations, although the interval between points was variable between individuals depending on light levels collected by the tag. The smallest interval between geolocation points was one day, and the largest interval was 29 days. The mean number of usable daily geolocation points (transmitted data) per individual track for northern tags was 35 (±7 points) (range 6 to 102 points) and for southern tags was 106 (±13 points) (range 7 to 190 points). Tags deployed in the northern group recorded data for a total of 3,729 days, from which 638 days (17%) had corresponding daily geolocations. The tags deployed in the southern group recorded 3,102 days total, from which 1,491 days had corresponding daily geolocations (48%). Total retention time (based on an expected 365 day program) was approximately 54% (northern) and 57% (southern), with seven of the tags lasting the full 365 day duration.

**Table 1:** Summary of spiny dogfish (*Squalus acanthias*) PSAT tag deployments and pop-up date, locations, and time at liberty.

Tag ID	Sex	Total Length (cm)	Tag Date	Tag Site	Pop-up Date	Days at Liberty	Pop-up Lat (°N)	Pop-up Long (°W)
96476	M	78	10/2/2009	GOM	3/13/2010	162	43.246	67.128
96477	M	80	10/2/2009	GOM	1/8/2010	98	41.165	68.567
96478	F	83	11/9/2009	GOM	3/9/2010	120	42.644	69.703
96479	F	89	10/2/2009	GOM	11/17/2009	46	43.131	68.916
96480	M	84	10/2/2009	GOM	10/2/2010	365	42.947	70.552
96481	F	87	10/2/2009	GOM	10/2/2010	365	41.749	70.328
96482*	F	86	10/2/2009	GOM	8/10/2010	312	43.463	70.373
96483*	F	91.5	10/2/2009	GOM	8/20/2010	322	42.017	70.115
96484	M	77	10/2/2009	GOM	6/5/2010	246	41.346	70.600
96485*	M	71	10/2/2009	GOM	10/3/2010	366	42.061	70.159
96486	M	NR	8/13/2010	GOM	11/30/2010	109	39.923	69.896
96487	F	100	7/17/2010	GOM			NON REPORTER	
96488	F	87.5	7/17/2010	GOM	2/28/2011	226	39.353	72.474
96489	M	NR	8/13/2010	GOM	11/10/2010	89	40.439	69.803
96490	F	84.5	7/17/2010	GOM	8/5/2010	19	42.362	70.196
96491	F	80	7/17/2010	GOM	10/13/2010	88	41.931	70.312
96492	M	NR	8/13/2010	GOM	9/9/2010	27	42.053	69.267
96493	M	68	8/13/2010	GOM	5/13/2011	273	39.085	72.367
96494	F	94.5	7/17/2010	GOM	11/25/2010	131	40.785	71.754
96495	F	89	7/17/2010	GOM	7/17/2011	365	41.789	67.652
97649	F	90	4/13/2011	NC	4/23/2011	10	35.523	75.071
97650	F	93	4/13/2011	NC			NON REPORTER	
97651	F	88	4/13/2011	NC	6/8/2011	56	37.453	70.064
97652	F	NR	4/13/2011	NC	3/22/2012	344	40.16	62.89
97653	F	92	4/13/2011	NC			NON REPORTER	
97654	F	99	4/13/2011	NC			NON REPORTER	
97655*	F	92	4/13/2011	NC	2/6/2012	299	35.14	73.79
97656	F	88	4/13/2011	NC	4/16/2012	369	38.027	74.042
97657*	F	83	4/13/2011	NC	7/7/2011	85	41.493	71.422
97658	F	91	4/13/2011	NC	12/28/2011	259	35.243	74.737
97659	F	90	4/13/2011	NC	10/4/2011	174	41.546	68.891
97660	F	93	4/13/2011	NC	1/17/2012	279	34.618	77.174
97661	F	104	4/13/2011	NC	10/8/2011	178	41.133	71.355
97662	F	94	4/13/2011	NC	6/15/2011	63	39.182	73.913
97663	F	87	4/13/2011	NC			NON REPORTER	
97664	F	97	4/13/2011	NC			NON REPORTER	
97665	M	74	4/13/2011	NC	8/20/2011	129	41.999	67.623
97666	F	NR	4/13/2011	NC	2/18/2012	311	38.005	67.654
97667*	F	101	4/13/2011	NC	12/3/2011	234	39.598	74.353
97668	F	NR	4/13/2011	NC	2/19/2012	312	42.694	45.438

*Denotes recovered and returned tag.

NR = “not recorded”.

**Figure 1:**
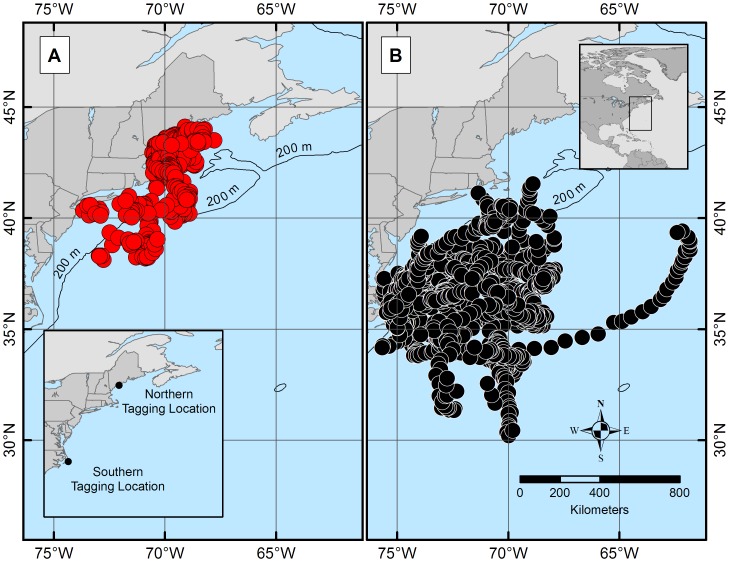
Northern aggregate geolocation points (A) span the area between Rockland, ME (∼44°N) and the Virginia/North Carolina line (∼36°N), with the majority (67%) between Rockland, ME and Cape Cod, MA and few (33%) south of Cape Cod. The majority (73%) of southern aggregate geolocation points (B) span the area between Albemarle Sound, VA (∼36°N) and New Smyrna Beach, FL (∼29°N), while the remaining points (27%) reach as far as Cape Cod. Tag deployment sites are marked in the Gulf of Maine (northern tags) and off the coast of North Carolina (southern tags).

**Figure 2:**
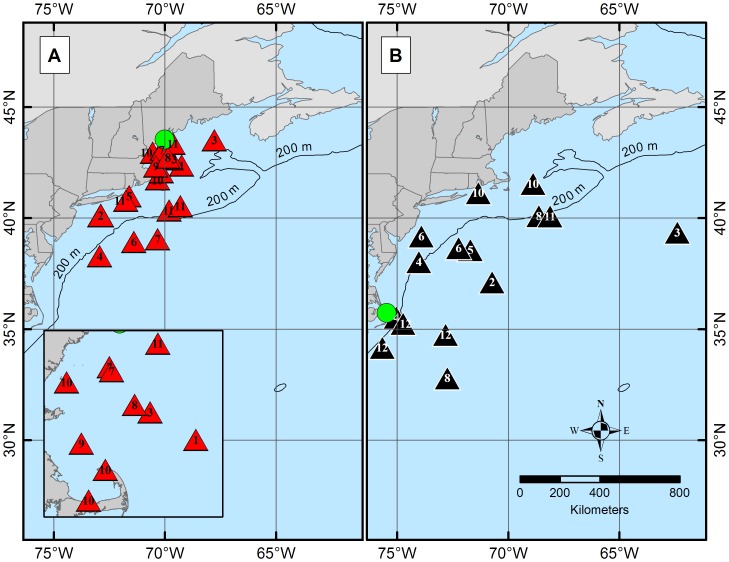
Pop-up points for northern (A) and southern (B) spiny dogfish tags. Each point represents one tag pop-up point. The numbers inside of the triangles represent the month in which the tag popped. The green circle represents the point of deployment for both the northern (A) and southern (B) tags. Estimated 95% and 50% UDs for northern (A) and southern (B) spiny dogfish tags. 95% UD or total home range (light red bounding area) for the northern dogfish extends from Maine to Maryland, while the 50% UD or concentrated usage core space (dark red central area) lies between approximately Rockland, ME and Cape Cod, MA. The red points are the geolocations used to calculate the UDs. 95% UD (light grey bounding area) for the southern dogfish lies between approximately Cape Cod, MA and Georgia, while the 50% UD (black central area) extends from the Delaware/Maryland line and Outer Banks, NC. The black points are the geolocations used to calculate the UDs.

**Figure 3:**
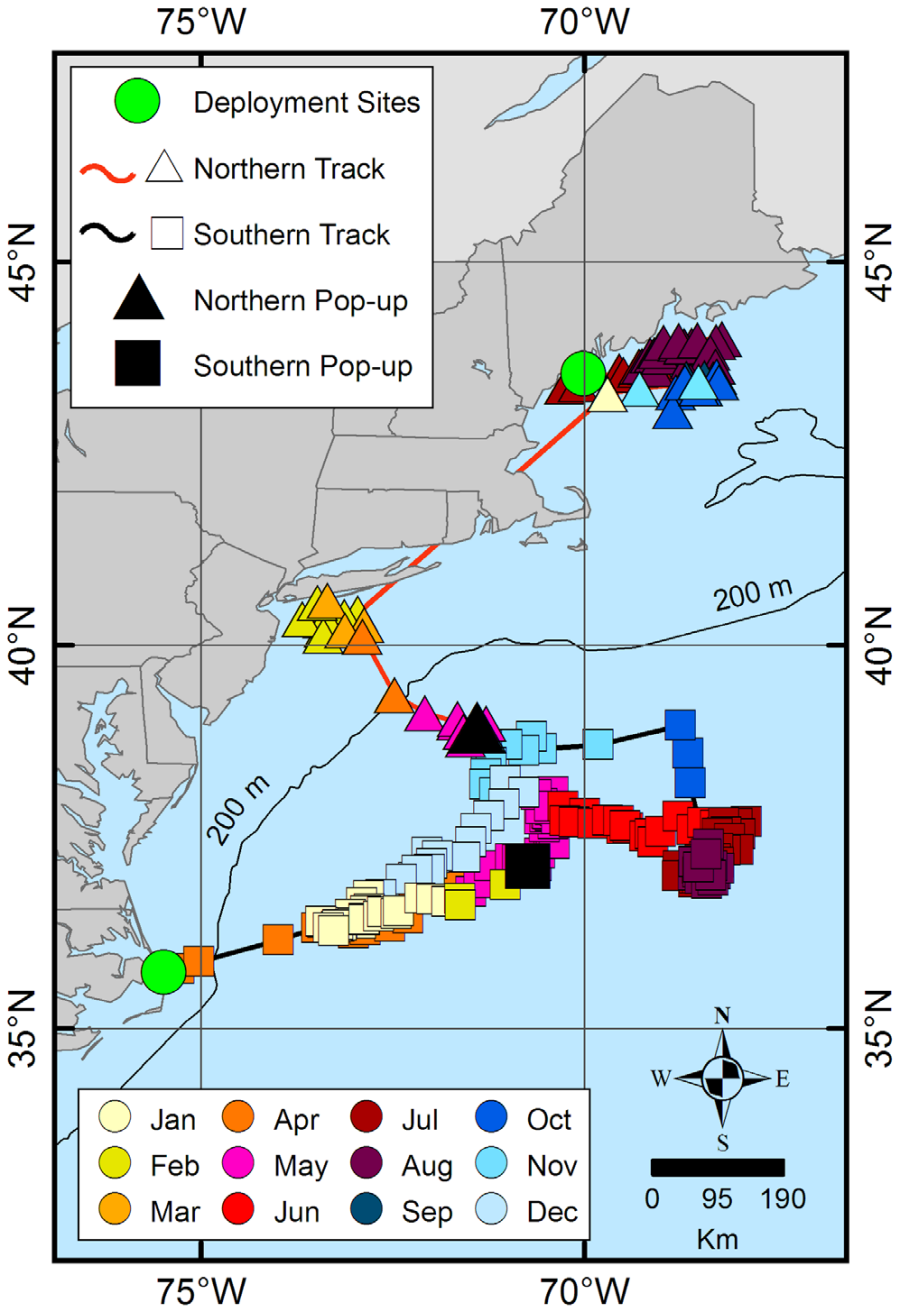
Two representative 365-day tracks for one northern (triangles) spiny dogfish and one southern (squares) spiny dogfish. Deployment sites (north = Maine, south = North Carolina) are indicated by green circles. Colors of points represent different months throughout the year.

**Figure 4:**
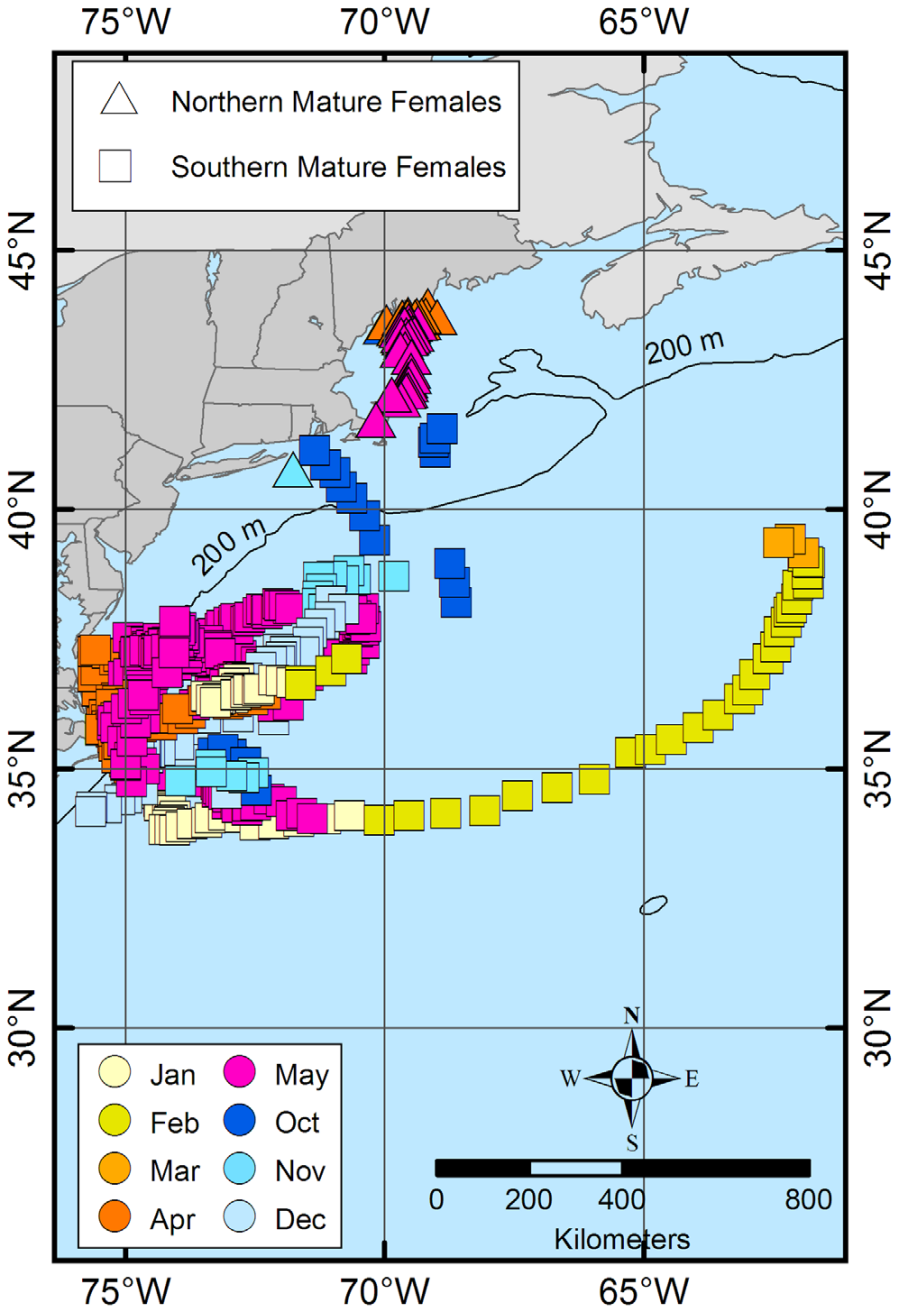
Geolocations for mature females (> = 88 cm) from both the northern (triangles) and southern (squares) tags. Colors of points represent different months of proposed parturition.

**Figure 5:**
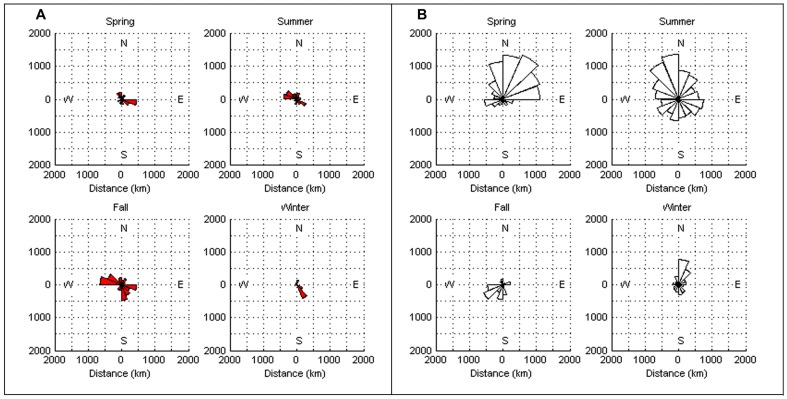
Circular (directional) seasonal histograms show the direction and magnitude of the majority of movement from individual geolocation points for the northern (A) and southern (B) tags.

The estimated daily geolocations (Figure 1A) and majority of known pop-up locations (Figure 2A) from the northern spiny dogfish revealed trends in movement patterns that appeared to be regionally centered. The majority of these geolocation points (67%) were located north of ∼42°N (Cape Cod, MA) and south of ∼44°N (Rockland, ME). The remaining points (33%) of points, primarily from one individual migrant shark (tag ID 96488), were located between Cape Cod, MA and ∼36°N (Virginia/North Carolina line). Conversely, the estimated daily geolocations (Figure 1B) and known pop-up locations (Figure 2B) for the southern spiny dogfish were more dispersed from the deployment site than the northern spiny dogfish, but do not extend into the GOM. Most of the southern daily geolocation points (73%) were located between ∼36°N (Albemarle Sound, VA) and ∼29°N (New Smyrna Beach, FL), while the remaining points (27%) span between Albemarle Sound, VA and Cape Cod, MA. Similar to the northern satellite tags, one southern spiny dogfish (ID 97652) deviated from the group majority of geolocations. Two representative tracks (one from each tagging group) are shown in Figure 3 from individuals that retained their tags for 365 days. When month specific geolocations for mature females in both regions were mapped, the majority of the geolocations were close to respective deployment sites (either in the GOM or off NC) with little overlap between the groups along the mid-coast (Figure 4). Seasonal circular histograms of individual movement by region suggests little to no distinct seasonal pattern in the northern geolocations, but stronger seasonal directionality for the majority of movement in the southern geolocations (Figure 5). The southern individuals show strong northeastward (spring) and northwestward (summer) movement of greater magnitude than the northern individual movements for the same seasons (eastward and northwestward majority movement respectively). Autumn and winter movement for the southern spiny dogfish was not as strong in magnitude, however the majority of movement does suggest a southwestward (autumn) and northeastward (winter) movement tendency. While the northern autumn (westward and southeastward) and winter (southeastward) movements are of lesser magnitude compared to the southern spiny dogfish, the summary of data still suggest strong movements within the northern region.

**Figure 6:**
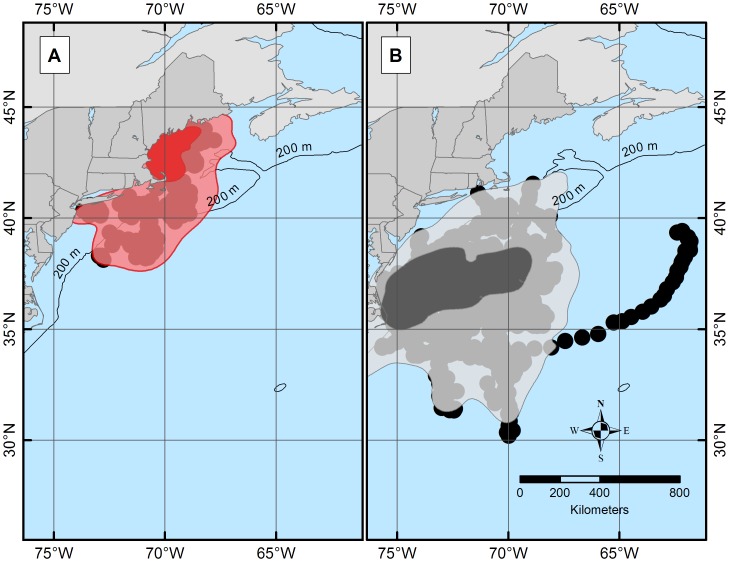
Estimated 95% and 50% UDs for northern (A) and southern (B) spiny dogfish tags. 95% UD or total home range (light red bounding area) for the northern dogfish extends from Maine to Maryland, while the 50% UD or concentrated usage core space (dark red central area) lies between approximately Rockland, ME and Cape Cod, MA. The red points are the geolocations used to calculate the UDs. 95% UD (light grey bounding area) for the southern dogfish lies between approximately Cape Cod, MA and Georgia, while the 50% UD (black central area) extends from the Delaware/Maryland line and Outer Banks, NC. The black points are the geolocations used to calculate the UDs.

When the aggregate geolocations from each region were used to estimate utilization distributions (UDs), both 95% and 50% UDs revealed distinct spatial and temporal patterns. However, since 50% UDs are more indicative of bulk movement, all ensuing analysis focused on the interpretation of that particular UD data [66], [76]–[78]. The 50% UD for the northern group highlighted an area in southern GOM close to shore between ∼41°N and ∼44°N, (Figure 6), whereas the 50% UD for southern spiny dogfish was centered between Delaware (∼38°N) and North Carolina (∼35°N).

**Figure 7:**
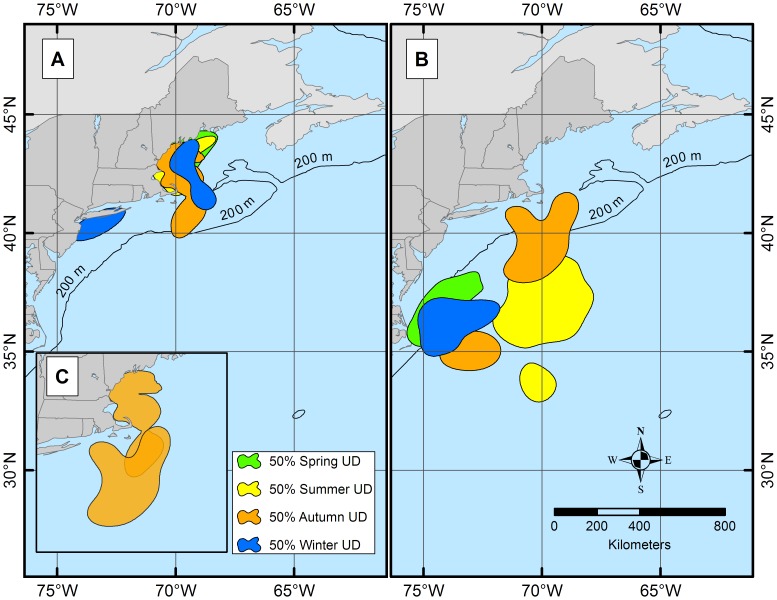
Seasonal 50% UDs for both the northern (A) and southern (B) deployed dogfish. The 50% spring UDs are represented in green, summer in yellow, autumn in orange, and winter in blue. Autumn was the only season that showed spatial overlap between the northern group and southern group (C).

Seasonal 50% UDs for the northern group remained in the same general area in the GOM (winter–summer) and showed no distinct seasonal movement pattern (Figure 7A). Northern UDs remained inshore (east of the continental shelf break) throughout all four seasons, expanding slightly more towards offshore (west of the continental shelf break) in autumn and winter. In contrast, seasonal 50% UDs for the southern group showed a different pattern, cycling in a slightly larger and clockwise inshore and offshore pattern (Figure 7B), which again was to be expected based off of the individual movement histogram results. Utilization distributions for the southern group remained inshore in winter and spring and expanded further offshore than the northern group in summer and autumn. When the seasonal 50% UDs were compared between regions, autumn was the only season with overlap (between Cape Cod and New Jersey) (Figure 7C). Plotting the NEFSC bottom-trawl survey stations with the temporally corresponding geolocations (with CIs) suggested spatial overlap (horizontal availability to the surveys) between the tagged sharks and the seasonal survey. Results indicate only 31.5% (autumn) and 56.1% (spring) of geolocations were horizontally available during the respective spring and autumn surveys. When broken down further into months which trawl surveys occurred, monthly horizontal availability was highly variable, ranging from 0%, where tag geolocations (with CIs) did not overlap with trawl survey areas at all, to 100% availability where all tag geolocations were located within the corresponding trawl survey areas. Seasonal vertical availability (from the points that were horizontally available), to trawl depths yielded 13.0% total availability overlap in spring and 12.7% total availability overlap in autumn. Monthly total availability fluctuated from 0–27.8% between October 2009 and April 2012.

**Figure 8:**
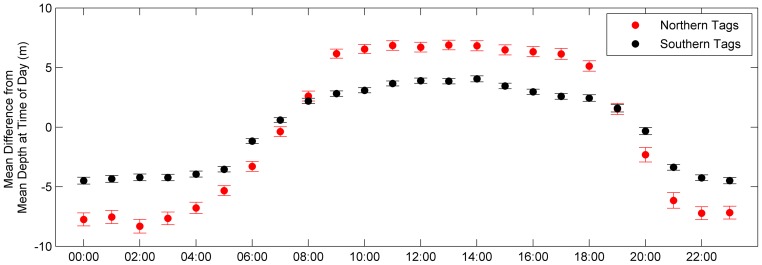
Mean differences between hourly diel depths and mean daily depths for the northern tags (red) and southern tags (black) to reveal distinctly different diel patterns. The northern tags show a larger change in mean diel depths than the southern tags. Error bars represent standard error.

Analysis of vertical movement patterns indicated both the northern and southern spiny dogfish actively utilized a large portion of the water column. Differences in depth ranges for the two groups were observed with the northern group occupying waters from the surface (0 m) to depths of 481.5 m, and the southern group occupying waters from the surface to depths of 214.5 m. The northern group primarily resided at an overall mean depth of 92.6 (±0.1 m), which was significantly different (t (8774) = 91.6, p<0.001) and almost three times as deep as the mean depth (26.9±0.2 m) occupied by the southern group. Both groups of spiny dogfish displayed significant diel patterns, with differences between the two groups (F (1, 1) = 92.1, p<0.001), occupying overall shallower depths (northern: 79.8±0.4 m, southern: 23.8±0.9 m) during night and overall deeper depths (northern: 100.1±0.4 m, southern: 25.4±1.0 m) during the day. Comparing the differences between hourly diel depths and mean daily depths suggests different diel patterns between the two groups, as the northern spiny dogfish displayed a more drastic change in depths from night and day than the southern spiny dogfish (Figure 8).

**Figure 9:**
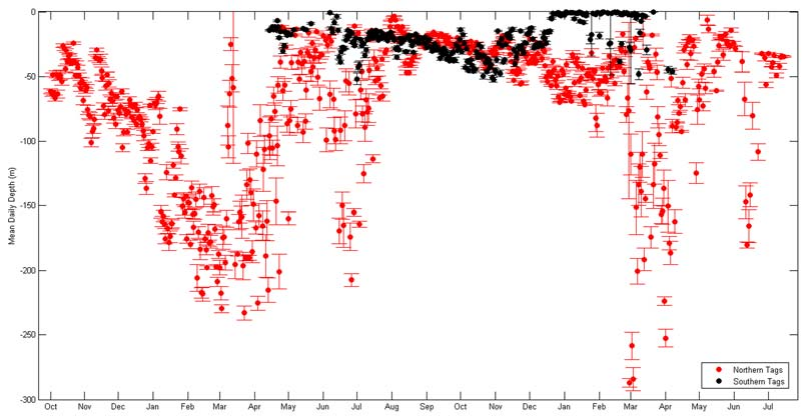
Mean daily depth time series for the northern (red) and southern (black) tags, ranging from 10/2/2009 to 7/17/2011 for the north and 4/13/2011 to 4/13/2012 for the south. Error bars represent standard error.

Additionally, seasonal depth results indicated substantial differences (F (1, 3) = 2801.1, p<0.001) for both groups (Figure 9). The mean depths for the northern group ranged from 41.7 (±0.2 m) in autumn, 89.5 (±0.4 m) in winter, 112.5 (±1.2 m) in spring, and 73.5 (±0.6 m) in summer. Similarly, mean seasonal depths for the southern group were also different, though they were less variable than the northern group. Southern sharks utilized deeper depths during summer (45.6±0.1 m) and autumn (37.6±0.1 m) and shallower depths in winter (7.9±0.1 m), and spring (24.1 m±0.1 m).

**Figure 10:**
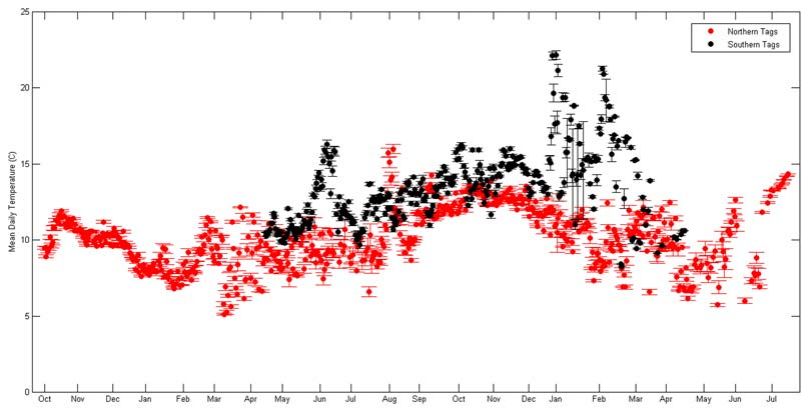
Mean daily temperature time series for the northern (red) and southern (black) tags, ranging from 10/2/2009 to 7/17/2011 for the north and 4/13/2011 to 4/13/2012 for the south. Error bars represent standard error.

The sharks tagged in both the northern and southern group inhabited a wide range of temperatures, but had considerably different (t (9110) = −35.7, p<0.001) overall average temperatures (Figure 10). Analyses of temperature data revealed that although spiny dogfish tagged in the northern group exhibited a large range in temperature (2.8 to 19.2°C), the mean overall temperature utilized by these sharks was 9.2 (±0.1°C). Similarly, the sharks from the southern group also exhibited a large overall temperature range (5.0 to 22.1°C) with a mean temperature of 12.7 (±0.1°C), resulting in a significant difference (t = 467.217, p<0.001) from the northern group. Seasonal differences in recorded temperatures were also observed (F (1, 3) = 484.8, p<0.001). The northern sharks appeared to oscillate from the warmest temperatures in summer (9.7±0.1°C), and autumn (9.5±0.1°C), progressively declining during winter (8.9±0.1°C), to its coolest mean in spring (8.8±0.1°C). More pronounced than the northern spiny dogfish, the southern sharks displayed a similar pattern with warmest temperatures in autumn (13.8±0.1°C), progressively declining in winter (13.7±0.1°C), to the coolest in spring (10.8±0.1°C), and warming again in summer (12.4±0.1°C). Differences between the mean seasonal temperatures between the sexes (northern tags only) never reached greater than 1°C.

**Figure 11:**
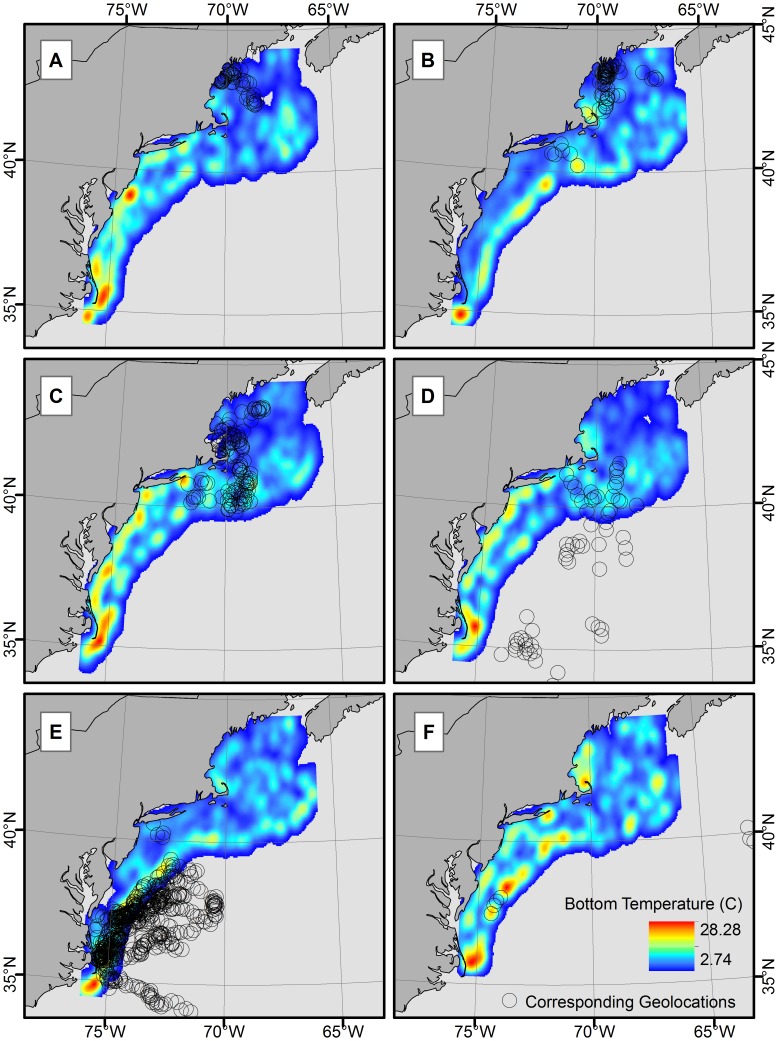
NMFS bottom-trawl data of survey station bottom temperatures for autumn (A. 2009, C. 2010, E. 2011) and spring (B. 2010, D. 2011, F. 2012) trawls overlaid with corresponding northern and southern geolocations of current study.

Comparisons of temperatures obtained from satellite tagged spiny dogfish to spatiotemporally corresponding bottom temperatures obtained from NEFSC bottom-trawl data suggested divergent results. Interpolated bottom temperatures gathered from the autumn surveys (bottom temperatures 9.0±0.1°C in the northern survey area, bottom temperatures = 14.8±0.1°C in the southern survey area) were within approximately 1°C of mean autumn temperatures obtained from the tagged sharks, 9.5±0.1°C (northern) and 13.8±0.1°C (southern) (Figure 11A, C, E). The NEFSC spring bottom-trawl temperatures averaged 6.5 (±0.1°C) in the northern survey area and 8.1 (±0.2°C) in the southern survey area (Figure 11B, D, F). Bottom temperature values were greater than 2°C cooler than mean tag temperatures for both groups, 8.8 (±0.1°C) (northern) and 10.8 (±0.1°C) (southern).

## Discussion

The large sample size of PSATs [79]–[80] utilized in the current study (n = 34), enabled the use of satellite tags to provide high-resolution information on the broad-scale movements, depth, thermal habitats, and survey availability of spiny dogfish in the northwest Atlantic Ocean. However, given the error around the estimated point geolocations, the general trends observed between the two tagging groups are intended to be indicative of only conservative, broad-scale movement patterns, as reported in other PSAT studies [5], [81]. With this in mind, the data herein indicates the bulk (67% and 73%) of each of the northern and southern geolocation points were segregated towards respective deployment sites and did not follow the previously described migration pattern for this species along the U.S. east coast [25]–[30]. When general movement patterns from the two groups were further modeled using aggregate geolocations and UDs these data provided a better overall sample-wide understanding than using individual track analyses. For instance, two-thirds of the geolocation points in each group were non-overlapping (with geolocations from the opposing group), indicating an apparent separation in the northern and southern groups within the spiny dogfish's known centralized range. Additionally, the two groups also had divergent movement patterns from one another, with the northern spiny dogfish staying more localized and less synchronous within the group, while the southern spiny dogfish were more widespread with more distinct synchronous oscillatory patterns. Despite the large, acknowledged error associated with these geolocation techniques [5], [81], the present data suggests there were differences in overall movement between the two groups.

The findings presented herein support previous movement patterns from satellite tagged spiny dogfish which observed restricted movement patterns in sharks tagged within the GOM [43] and previous TRAC Status Report findings, suggesting overall segregated spatial structuring between the northern and southern extents of their known range [36]. While other reports from bottom trawl surveys [82]–[87] and conventional tagging studies [8], [88] suggest broad-scale movement patterns and aggregations of spiny dogfish in the GOM during autumn and along the shelf off NC during spring, these findings were not supported by the data herein. This could possibly be explained by relatively low sample size (compared to the U.S. east coast population as a whole) and limited spatiotemporal tagging events (four in the northern site, one in the southern site). Due to the timing of the northern tagging events, we cannot rule out the possibility that some of the sharks tagged had already completely migrated north and thus stayed relatively close to the tagging site. The lack of variation in spatiotemporal events could also affect the apparent deficiency of northward movement if migrating individuals had already moved into the northern most extent of the seasonal migration pattern. For example, if spiny dogfish had already left the tagging site area in the north (i.e. moving further north in the GOM) prior to the tagging events, those tagged individuals could likely be non- or lesser migrating residents, making a less northward trip that late in the migration season.

A notable observation in the study was the segregation of the northern and southern groups of spiny dogfish. A comparable segregation behavior has been described in tagged manta rays (*Manta birostris*) [89] in the southern Gulf of Mexico, suggesting tagged individuals in some areas of a population remained resident (<116 km away from tagging site) much like the northern spiny dogfish, while rays in other areas made long distance migrations, similar to the southern spiny dogfish. The same behavior has also been observed in salmon sharks [20] and spiny dogfish (*Squalus acanthias*) in the Pacific Ocean [90]. Additionally, spiny dogfish within both regions of the current study displayed resident (individuals that remained within the 2/3 bulk geolocation aggregations closest respective tagging sites) and migratory (individuals that traveled into the 1/3 geolocation overlap) movement. This resident and migrant behavior within a population has been observed in white sharks in Hawaii [91] and brown trout (*Salmo trutta*) in Norway [92]. For instance, the bulk movement in autumn and winter (northern group) and summer and autumn (southern group) suggested divergence in aggregate geolocation, and convergence of geolocations for both groups overlapping in autumn. These separations within each group could indicate the parting of a breeding stock (migratory individuals from both north and south), that congregate near Cape Cod in autumn, and a non-breeding stock (resident individuals) from each group that stays closer to respective deployment sites. This same complex pattern of breeding and non-breeding stocks was observed in white sharks around Guadalupe Island, Mexico where a proposed two year migration pattern based off an 18-month gestation was driven by a mating route (year 1) and a non-mating route (year 2) [24]. The same population of white sharks in Mexico also exhibited analogous patterns of a primary area of seasonal overlap between two subpopulation groups [22], suggestive of a possible site for prime foraging or mating [23] and similar to the patterns seen herein, based off of what is known about spiny dogfish gestation [45]–[46], [59], [61].

The reproductive mode of the spiny dogfish is characterized as yolk-dependent viviparity in which full embryonic development occurs within the uteri of the mother, and a yolk-sac provides the majority of the nourishment [38]. Follicular maturation and gestation occur simultaneously in this species, once females reach sexual maturity [93]. Although females were not assessed for reproductive status in the current study, all females were mature and assumed gravid [38]. Previous research suggests that parturition and/or mating [29], [45] in spiny dogfish occurs inshore from late autumn to late winter in the U.S. population [59], [62], [63]–[65]. Those findings generally spatiotemporally coincide with autumn (northern and southern) and winter (northern) 50% UDs [65] and could explain why the groups were found to converge during this time. If indeed this is the case, geolocations of mature females during the proposed pupping season (October–May) support previous studies which suggest both inshore and offshore pupping areas exist for the US population [26], [59]–[60], [62]–[65]. However, due to the limitations above and the temporal difference between the two groups, further research needs to be conducted on how gestation period and movement patterns may be connected before any conclusions can be drawn.

The majority of the satellite tags deployed in the northern region remained in coastal waters of the GOM during spring (2010–2012), suggesting that not all spiny dogfish migrated southward, and were therefore not accounted for during those particular seasonal surveys. This finding also contradicts previous documented movement patterns [26]–[30], which suggest that spiny dogfish should be absent from the GOM during this time of year [31]. Results of the current study demonstrate movement outside (offshore) of the NEFSC bottom-trawl survey area and a high degree of vertical activity, suggesting that spiny dogfish captured by bottom-trawls could represent a smaller proportion than previously thought, resulting in potentially underestimated biomasses [94]–[95]. It is possible that spatial constraints (west of the continental shelf break, selective substrate types), a single gear type (bottom-trawl), and temporal restrictions (limited to two seasons per year, starting of the coast of NC and moving north to the GOM) may in part, be responsible for the variability in the NEFSC survey biomass estimates relative to the true population of dogfish [82]–[87]. In addition, vertical distributions of many species vary with time of day, affecting the availability of fish to demersal trawl gears [96]. Frazier et al. [97] suggested that trawl survey estimates of catch density need to be converted to estimates of actual density by taking into account the catchability of the fish involved in the particular gear employed. When a catchability coefficient was applied to North Sea bottom groundfish species, the catch correction suggested that raw trawl survey density data significantly underestimated actual densities [97]. Based on this new information pertaining to spiny dogfish seasonal horizontal and vertical distribution and the results of Frazier et al.'s study, a modified correction factor for dogfish abundance estimates could provide insight into the observed variability in biomass of this species (i.e. q, gear availability).

Pop-up archival satellite tags have expanded the knowledge of vertical and thermal habitat preference previously unknown for spiny dogfish and other species. The behavior observed in the current study suggests that spiny dogfish vertical activity is not representative of a predominantly benthic species [98]–[99], and that this species actively uses the majority of the water column throughout the day, corroborating the findings of Sulikowski et al. [43]. Similarly, satellite tags have revealed behavioral patterns in immature Greenland sharks (*Somniosus microcephalus*),suggesting this species spends a considerable amount of time off the bottom and depth preferences appear to be similar to a more pelagic shark species [100]. In other species, previously undocumented vertical utilization of tagged juvenile Atlantic bluefin tuna (*Thunnus thynnus*) was observed in the northwest Atlantic Ocean, providing valuable insight for assessing stock structure and fisheries plans [101].

In the current study, the mean depth utilized by the southern group (26.9 m) was less than half of the mean depth of the northern group (92.6 m), which could be attributed the southern group being limited geographically to shallow depths, (west of the shelf edge, south of Virginia; maximum depth approximately 150 m) compared to the northern group (west of the shelf edge, north of Cape Cod; maximum depth approximately 450 m). However, since sharks within the southern group traveled further off the continental shelf and into much deeper water than the northern group, the data herein would suggest the southern group was not constrained to shallow water, but possibly seeking a preferred depth.

Overall vertical activity peaked in different seasons for the northern (summer) and southern (winter) groups. Within this high amount of vertical activity, the observed seasonal mean depths between the two groups suggest non-synchronous cyclic patterns in depths utilized. The two groups were closest to each other during autumn, with a mean depth of 41.7 m (northern) and 37.6 m (southern), corroborating the horizontal movement data, which suggests a possible overlap in northern and southern spiny dogfish habitat for this season. Similar to Sulikowski et al. [43], strong oscillatory diel behaviors were also observed, where spiny dogfish inhabited deeper waters during daytime and shallow waters during nighttime. This pattern of vertical movement has been observed in other elasmobranch species such as white sharks [102], scalloped hammerheads, (*Sphyrna lewini*) [103], sixgill sharks (*Hexanchus griseus*) [104], bigeye threshers (*Alopias superciliosus*) [105], and basking sharks (*Cetorhinus maximus*) [106], where the pattern has been linked to prey searching or locating optimal temperature and oxygen conditions [106]–[107]. Considering the narrow range in seasonal temperature fluctuations (less than 1°C) within both groups, and known opportunistic predation tendencies [28]–[29], it is possible the spiny dogfish are behaviorally seeking out optimal temperatures at varying depths in response to seasonal temperature changes [108]–[110] or changing position in the water column in response to prey availability, driving the dive oscillations. While the reasons behind the observed patterns cannot be elucidated from the current study, similar behavioral patterns have been observed in other species. For example, blue sharks [98], bluefin tuna [111], shovel-nose guitarfish [112] and leopard sharks [113] are thought to seek out ideal temperatures or relatively small temperature ranges to optimize daily metabolic and foraging requirements.

In addition to the prevalence for seeking an optimal temperature, the differences in mean temperatures between the northern and southern groups corroborate the segregated horizontal movement patterns observed in the current study. The apparent tolerance for large ranges in temperature also suggests that the population of tagged spiny dogfish in the current study have no need to make seasonal latitudinal migrations to warmer and/or cooler waters as was once thought [8], [29]. This type of pattern is supported by Fisk et al. [100] who suggested a high temperature tolerance and increased temperature range could alter a species' geographic preferences. This phenomenon has been observed in a number of species in the U.S. portion of the northwest Atlantic where both northward shifts (i.e. alewife (*Alosa pseudoharengus*), American shad (*Alosa sapidissima*), silver hake (*Merluccius bilinearis*), red hake (*Urophycis chuss*), and yellowtail flounder (*Limanda ferruginea*)) and expansion in area occupied (i.e. winter skate (*Leucoraja ocellata*), Atlantic herring (*Clupea harengus*), spotted hake (*Urophycis regia*), winter flounder (*Pseudopleuronectes americanus*), and Atlantic mackerel (*Scomber scombrus*)) [114]–[115] are likely a causal factor in the observed movement patterns of the dogfish in the current study.

The information obtained in the current study from satellite tagged sharks appeared to have reduced some of the uncertainties associated with spiny dogfish vertical and horizontal movement, while also providing insight into the potential availability of this species in NEFSC bottom-trawl surveys [43], [116]. Several factors can affect the likelihood of availability in a bottom-trawl survey, including the uncertainty of horizontal migration patterns, environmental influences, and degree of vertical activity of the target species [94], [97]. In general, availability was extremely variable and inconsistent from month to month, ranging from low (0%) to high values (100%). However, the overall low total availability of spiny dogfish (highest estimate 27.8%) combined with an observed temperature difference of greater than 2°C between the tags (± tag error) and bottom-trawl data, suggests that vertical position in the water column of the spiny dogfish and the trawl net are likely not in proximity to one another for at least some portion of the year. Regardless, the availability results presented herein suggest a large portion of the spiny dogfish population is most likely not sampled on an annual basis in the NEFSC bottom-trawl survey.

## Conclusions

The observed differences between the two tagging groups of spiny dogfish on the U.S. east coast in utilization space and overall movement patterns are not indicative of a migration pattern associated with single coast wide populations of large-scale migratory species. In addition, the results suggest that the estimated spiny dogfish movement patterns calculated from satellite tag data are possibly spatiotemporally asynchronous with the NEFSC bottom-trawl surveys thus, a potentially large percentage (horizontal and vertical “availability”) of these sharks may be unaccounted for in this survey. Further investigations are needed to address the observed differences in habitat utilization, availability and stock structure to determine the degree of segregation, and possible additional subpopulations or metapopulations, between the northern and southern spiny dogfish and to augment future management plans.
